# Neuromodulation of the Posterior Tibial Nerve for the Control of Urinary Incontinence

**DOI:** 10.3390/medicina58030442

**Published:** 2022-03-17

**Authors:** Álvaro Astasio-Picado, María García-Cano

**Affiliations:** Nursing, Physiotherapy and Occupational Therapy Department, Faculty of Health Sciences, University of Castilla-La Mancha, Real Fábrica de Sedas, s/n, 45600 Talavera de la Reina, Toledo, Spain; maria.garcia@alu.uclm.es

**Keywords:** urinary incontinence, neuromodulation, tibial nerve, overactive bladder, transcutaneous, percutaneous, stimulation

## Abstract

Urinary incontinence is considered a health problem that both elderly and young people can suffer, most often elderly women. This problem can lead to difficulties in establishing social relationships and dependence, negatively affecting the quality of life of the people who suffer from it. To evaluate and analyze the studies that demonstrate the efficacy of interventions based on the neuromodulation of the posterior tibial nerve as a treatment for the control of urinary incontinence. The search period for articles focused on those published between March 2011 to March 2021, in five databases (Pubmed, Cochrane Library, Scielo, Google Academic and WOS) based on the clinical question, using the keywords derived from the DeCS and MeSH thesauri, combined with the Boolean operators “AND”, “NOT” and “OR”. The search was limited to publications from the last 10 years, in English and Spanish. After applying the selection criteria and evaluating the quality of the methodology, 5.28% (*n* = 27) of the 511 results were included with filters: 9 systematic reviews, 10 cohorts and 8 randomized controlled trials. After comparing the different articles, it was found that percutaneous stimulation of the tibial nerve is a suitable technique for treating overactive bladder. It is a promising technique in case of pelvic floor dysfunctions and effective for the control of urinary incontinence.

## 1. Introduction

The inability to control urination and an urgent feeling to go to the bathroom, known as urinary incontinence (UI), is considered a very important health problem that affects both young and old people, although to a greater extent women of advanced age. It is a symptom behind which there are multiple pathophysiological mechanisms that trigger a large number of hidden diseases. UI has a considerably negative impact on the quality of life of patients who suffer from it, since it can lead to dependence and difficulty in social relationships [1].

For this reason, it is very important to carry out a correct anamnesis, physical examination and complementary tests that are able to determine an adequate diagnosis, identifying the type of UI that the patient suffers, and thus, in this way carry out the correct treatment in order to resolve the causative pathologies and reduce the number of UI episodes. Nurses are considered to be the most appropriate professionals to carry out an assessment and continuous monitoring of the patient with UI, since it is a health problem with a nursing diagnosis of its own [2].

### 1.1. Urinary Incontinence

UI is the inability to control the loss of urine, frequently and distressingly, which may be by urethral or extraurethral route [1,2,3,4]. UI is a disorder that appears more frequently in women, as well as in geriatric people and, in addition, its prevalence increases with age [5]. Within the UI, two of the most frequent types stand out. With urge urinary incontinence (UUI), the patient cannot control the imminent urge to urinate. On the other hand, stress urinary incontinence is the loss of urine that occurs involuntarily after performing some physical effort, such as laughing, coughing or sneezing [4,5]. Some women may experience mixed UI; therefore, they present stress urinary incontinence together with UUI [5].

The International Continence Society designates overactive bladder syndrome (OAB) as “urinary urgency, usually accompanied by increased urinary frequency and/or nocturia, with or without urinary incontinence (wet overactive bladder; dry overactive bladder), in the absence of urinary tract infection or other detectable disease” [6,7].

### 1.2. Pathophysiology of Urinary Incontinence

In order to understand the functioning mechanism of continence and urination, it is necessary to consider that the bladder, from the physiological point of view, is an organ constantly functioning through a cycle of two phases, a voiding phase and a continence phase, which occur by visceral (parasympathetic and sympathetic) and somatic innervation [1,7].

However, it is important that the continence phase lacks bladder contractions, makes correct accommodations and closes the sphincter properly. Regarding the voiding phase, it is necessary to produce contractions of the detrusor muscle and the opening of the sphincter. To carry out these functions, visceral innervation, both sympathetic and parasympathetic, and somatic innervation are necessary, with integration in levels, firstly, at the medullary level, the midbrain level and finally the cortical level [1,8].

### 1.3. Neuromodulation

Direct neuromodulation of the sacral roots: It is a minimally invasive and reversible treatment. It consists of the direct stimulation of one of the different sacral roots, generally the third, for which it is named as neuromodulation S3. The technique is based on the introduction of an electrode through the sacral foramen S3, said electrode is responsible for sending electrical impulses to the root of the S3 nerve transcutaneous. The impulses sent are generated by an electrical impulse generator, which works by means of the energy provided by a battery. It is possible to regulate the intensity, pulse rate and width. In addition, it is a technique performed on an outpatient basis and under local anesthesia. It is considered important that before placing the stimulation box definitively, one is placed temporarily and externally so that the effectiveness of the treatment can be verified for a couple of days. This allows you to check whether the test is effective through symptoms, urination schedule and quality of life. Therefore, if the symptoms reappear when the test is stopped, it is considered a good criterion to implant the stimulation box. Neuromodulation S3 is considered a good alternative in patients with overactive bladder who have failed different pharmacological treatments [8,9,10,11,12].

Neuromodulation of the tibialis posterior: Neuromodulation of the external popliteal sciatic is a non-invasive technique. First, two electrodes are placed in the path made by the posterior tibial nerve; these electrodes can be self-adhesive or needle electrodes. Said electrodes are linked to a box located externally, which is capable of stimulating the path of the external popliteal sciatic through impulses with a controlled amplitude and frequency. To locate the posterior tibial nerve stimulation site, it is located behind the medial border of the tibia and 5 cm above the medial malleolus. Each session lasts approximately 20 min and it is recommended to carry out one or two sessions per week. In order to verify the effectiveness of the neuromodulation of the external popliteal sciatic, it is necessary for the patient to undergo treatment for at least 1 month. Neuromodulation of the external popliteal sciatic is effective in patients with bladder overactivity of both neurological and non-neurological origin [3,11].

The general objective of this work, through a systematic bibliographic review, is to evaluate and analyze the studies that demonstrate the efficacy of interventions based on neuromodulation of the posterior tibial nerve as a treatment for the control of urinary incontinence.

## 2. Materials and Methods

The preparation of this work was carried out through a systematic bibliographic review of the articles found by searching the following databases: Pubmed, Cochrane Library, Scielo, Google Academic and WOS. To find the best possible scientific evidence, a series of inclusion and exclusion criteria were applied.

Keywords for this review were: urinary incontinence, neuromodulation, tibial nerve, overactive bladder, transcutaneous and percutaneous stimulation. These have been validated by DeCS and MeSH. Once selected, the corresponding Boolean operators were used: AND/OR, as well as the necessary parentheses and quotation marks. The criteria that have been taken into account for the selection of the relevant studies are the following. Inclusion criteria: the search period for articles focused on those published between March 2011 to March 2021; studies based on interventions performed with neuromodulation of the posterior tibial nerve; studies aimed at the target population, that is, people between 30 and 90 years old; studies addressing urinary incontinence prevention issues; the selected studies must have scientific evidence and be published in corroborated databases; Spanish or English language. Exclusion criteria: articles prior to 2011; language: neither English nor Spanish; studies in which the population was younger than 30 years old and older than 90 years old; studies that do not provide scientific evidence justified by the level of indexing of articles in journals according to the latest certainties.

For the methodological evaluation of the individual studies and the detection of possible biases, the evaluation is carried out using the “PEDro Evaluation Scale”. This scale consists of 11 items, providing one point for each element that is fulfilled. The articles that obtain a score of 9–10 points will have an excellent quality, those between 6–8 points will have a good quality, those that obtain 4–5 points will have an intermediate quality and, finally, those articles that obtain less than 4 points will have a poor methodological quality [12].

The Scottish Intercollegiate Guidelines Network classification will be used in the data analysis and assessment of the levels of evidence, which focuses on the quantitative analysis of systematic reviews and on the reduction of systematic error. Although it takes into account the quality of the methodology, it does not assess the scientific or technological reality of the recommendations [13].

## 3. Results

**Figure 1:**
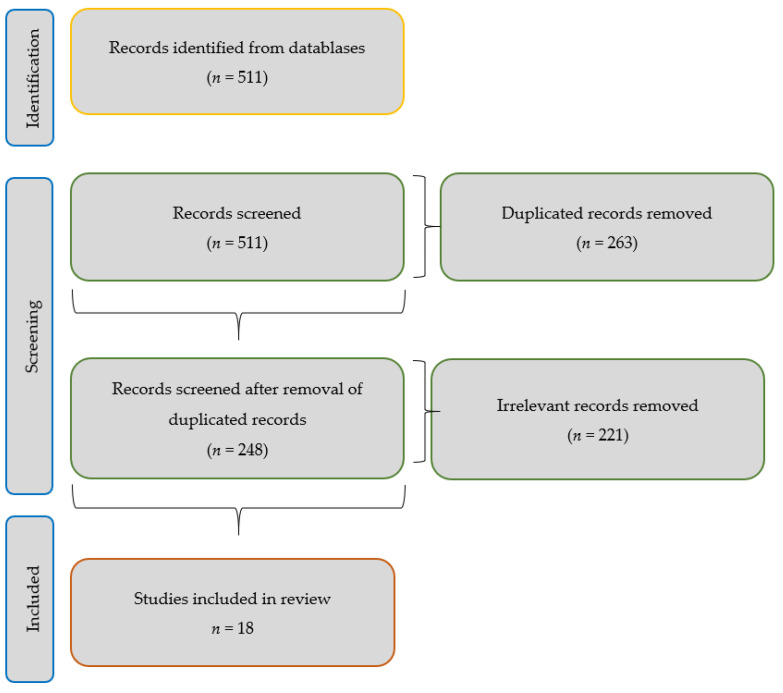
Flow diagram.

The research question was constructed following the PICO format (Population/patient, Intervention, Comparator and Results/Results). Detailed as “Adults of both sexes aged between 30 and 90 years (P), Neuromodulation of the posterior tibial nerve (I), Not compared (C), Reduction and control of UI (O)”. In the identification process, screening will be carried out according to the inclusion and exclusion criteria, with the aim of obtaining the final results of the study (Figure 1).

Below is a table that shows the search strategy used to select the 27 selected articles (Table 1). The total number of valid articles is summarized in Appendix A.

Posterior tibial nerve stimulation is an option in the management of OAB. Restarting treatment is indicated 24 months after the end of initial therapy [14].

### 3.1. Treatment with Percutaneous Stimulation of the Tibial Nerve

Treatment of OAB with percutaneous tibial nerve stimulation (PTNS) is also effective. This treatment is comparable to the effects produced by antimuscarinic treatments, but with greater safety in terms of side effects [14,15], and with better symptom control [16]. Women on PTNS for OAB achieve significant symptom relief within 2 years [17]. PTNS is a safe and effective technique for patients with OAB [18].

PTNS is effective for UUI. However, more studies are needed to improve the evidence on PTNS in nocturia and emergencies [19]. Posterior TN stimulation is currently one of the most promising therapeutic techniques for pelvic floor disorders [20]. Additionally, PTNS is one of the safest and most effective treatment options for neurogenic OAB after stroke [21]. PTNS is a minimally invasive process, highly effective in the treatment of OAB and pelvic pain. It also has few side effects, although it is limited, as patients must visit the office weekly for sessions [21,22].

### 3.2. Therapeutic Treatment with Sacral Neuromodulation

The therapeutic option for sacral neuromodulation is carried out using an implanted device capable of stimulating the S3 nerve root and, thus, improving pelvic pain, non-obstructive urinary retention and OAB symptoms [22]. Botulinum toxin A is recommended as third-line treatment for OAB (US) and for UUI (US and Europe) in patients who do not respond positively to drug therapy [22,23]. Before opting for surgery, it is necessary to evaluate all available treatment alternatives for OAB and UUI [23]. Tibial nerve stimulation may be safe and effective as a treatment for neurogenic lower urinary tract dysfunction and in patients with multiple sclerosis [24,25].

### 3.3. Treatment with Percutaneous Tibial Nerve Stimulation and Extended-Release Oxybutynin

PTNS and extended-release oxybutynin demonstrate similar benefits in OAB patients after 12 weeks of treatment [26]. PTNS is a neuromodulation technique used to facilitate bladder storage and modulate its function [27]. PTNS is an effective and well-tolerated treatment for UUIs that do not respond adequately to first-line therapies, and therefore, should be offered earlier in the treatment strategy [28,29]. PTNS can improve OAB symptoms for up to 24 months after treatment ends. In addition, the first sensation of fullness of the bladder and the diurnal urinary frequency act as independent predictors and are of great importance for the success of the PTNS [30]. Additionally, patients with multiple sclerosis and treated with PTNS can achieve significant durability for 12 months [31,32,33,34]. PTNS is a therapy that can provide both subjective and objective improvements for patients who do not respond to treatment with OAB medication [35,36,37,38,39,40].

## 4. Discussion

PTNS is a novel technique evidenced by the articles contrasted in this review where it is considered an adequate method for the treatment of OAB [14]. This technique is comparable to the effects of antimuscarinics, but with greater safety in terms of side effects [14,15]. To recommend PTNS as a treatment, long-term data and economic health analyses need to be obtained, as the included studies only estimated short-term outcomes after initial treatment in the review [15]. With a mean of 1.3 sessions per month, PTNS is a safe option for the treatment of OAB, as well as being long-lasting and effective in the long-term control of OAB symptoms [16]. Women on PTNS for OAB achieve significant symptom relief within 2 years. Therefore, it is considered a second-line treatment, safe and of excellent durability [17]. The PTNS is a safe and effective technique for patients with OAB, more studies are necessary to be able to evaluate the role of the PTNS in the rest of the situations and to be able to evaluate the durability of the treatment in the long term [18]. Furthermore, it is one of the most promising techniques for correcting and controlling pelvic floor disorders [20].

On the other hand, most of the selected articles conclude that it is a technique that is easy to apply [27,33], safe, effective in patients with OAB [15,16,17,18] and UUI [19] and of long duration [17]. In addition, it is also considered effective and safe in the case of neurogenic bladder after stroke [21]. Additionally, in patients with neurogenic lower urinary tract dysfunction [24] and in patients with lower urinary tract symptoms secondary to sacral modulation [25].

It is considered an invasive application technique for the control of OAB and pelvic pain [22,27,38]. However, for the sessions to take place, it is necessary for the patient to attend a consultation, which requires time [27]. This is considered a negative or risk factor, since the patient must visit the health center weekly. For this reason, implantable devices can be considered a good option to reduce the economic burden of PTNS in the long term [27]. In addition, a 6-week treatment provides greater access and cost-effectiveness to patients [40].

The average number of sessions for PTNS to be effective in OAB is 1.3 sessions per month. Although other authors conclude stating that the mean number of sessions that the patient must perform is 8.42 sessions per year and that the time between each session should be 64.3 days [17]. However, other articles consider that it is necessary to carry out 2 sessions per week, for 12 weeks with a duration of 30 min per session [26]. While other investigations consider that a single session per week is effective for 12 weeks with a duration of 30 min per session [33,36]. In the case of patients with PTNS as a treatment for post-stroke neurogenic bladder, the recommended guideline at the time of carrying out the sessions is 2 sessions per week for 3 weeks and with a duration of 30 min per session [21]. Another contribution indicates that 12 sessions are sufficient to achieve efficacy in UUI; however, once the 12 sessions have been carried out, this efficacy decreases without reaching the initial levels [39].

According to the author Pincus, it is advisable to give a reminder session per month for at least 1 year, since patients with reminder treatment continue to improve [36]. Continuous treatment reduces symptoms; however, these symptoms are similar after 6 weeks of initiation of treatment to those that can remain after 2 years with treatment [17]. On the other hand, Marchal proposes that the appropriate time to start retreatment in patients with OAB is 24 months after completing the initial therapy [14].

In most of the selected articles, it is observed that PTNS is a technique that, after being treated with it, decreases the daytime frequency [17,29,33], nighttime frequency [17,19,29,34], UUI episodes [17,19,29,33,34,35] and voiding frequency [19,34,35], increases quality of life [29,33], produces improvements in quality questionnaires [26] and increases voiding volume and emergency perception [29]. In the case of patients with post-stroke neurogenic bladder, the frequency and urgency of urination decrease and the data reflected in the voiding chart improve [21], while in sacral modulation patients, on the one hand, it produces an increase in voiding volume and, on the other, a decrease in urinary volume, urinary frequency, nocturia, UUI, urination/day and emergency episodes [31].

Regarding the adverse effects caused by PTNS therapy, only Yoong mentions an adverse effect discovered in one patient, which consisted of toe hypoesthesia lasting 4 months [17].

Yoong states that PTNS should be used as a second-line treatment with which symptom relief will be achieved at 2 years [17]. Additionally, Del Río S. explained that the improvement in symptoms lasts 24 months after completing the treatment with PTNS [30]. On the other hand, in patients with sacral modulation, the positive effects obtained last 12 months [31]. Furthermore, according to Iyer, patients who continued drug therapy after PTNS treatment did not see further improvements [34].

In some of the articles included in this narrative review, the authors make a comparison between the different treatment modalities for the control of UI, as is the case for Burton, who made a comparison between PTNS and antimuscarinic treatment. He obtained improvements with PTNS comparable to those obtained with antimuscarinics; however, he has greater safety with respect to adverse effects [15]. In the comparison between PTNS, sacral nerve stimulation and botulinum toxin A, PTNS has long-term effects and is not intended to achieve a motor response, but rather a sensitive response. In the case of Botulinum toxin A, it is effective with a single dose of 360 U every 3 months, which is why it is the most widely used as a third-line treatment in the US for OAB and UUI and in the case of Europe for IUU [23].

According to the author Manríquez, after making a comparison between treatments with PTNS (2 weekly sessions for 2 weeks with a duration of 30 min per session) or with 10 mg of prolonged-release oxybutynin/day, the benefits obtained are similar [26]. However, in another study by Vecchioli, a comparison is made between PTNS and sacral stimulation + pelvic floor muscle training. Although the results obtained are similar and the improvements are remarkable, only the perception of urgency increases in patients treated with PTNS [29]. Vecchioli compared the effectiveness of treatments with solifenacin, PTNS and PTNS + solifenacin, all of which were shown to be effective. However, PTNS showed greater efficacy compared to solifenacin, but when combining PTNS + solifenacin, greater effectiveness and durability than both techniques alone [32]. In the comparison of PTNS and chronic tibial nerve stimulation, better results are obtained with chronic tibial nerve stimulation, as it is safe, effective and requires fewer weeks of treatment [37], whereas in the comparison of PTNS and placebo, PTNS is more successful and effective [40].

Ramírez conducted the first randomized controlled trials, comparing PTNS and transcutaneous tibial nerve stimulation to assess their efficacy. Although there is no difference between the two in terms of obtaining results and they have no adverse effects, the prescription of transcutaneous stimulation of the tibial nerve may be increased due to its easier application compared to PTNS [33]. Furthermore, Valles concludes that performing 10 PTNS sessions per week lasting 30 min each is a well-tolerated and effective treatment that lacks negative effects in patients who do not respond adequately to first-line therapies [28].

It is vitally important before undergoing surgery to evaluate all available treatment options [23]. Urogynecological surgery is considered a negative factor for the improvement of patients [36]. Alka recommends the use of PTNS as a third-line treatment when pharmacological treatment has failed and reviewed by a multidisciplinary team [38]. A history of anxiety, depression and severe UUI are recognized as positive predictors of a successful outcome [35].

Priyanka determines that PTNS is the most widely used treatment for OAB and pelvic pain along with sacral neuromodulation [22]. It is also a technique that can provide both subjective and objective improvements in patients who have failed pharmacological treatments [34].

Many studies have concluded that the data obtained are not sufficient to select patients, duration of treatment, modality, long-term adverse effects [15,18,19,24,25,28,37] and the effects in other situations such as non-obstructive urinary retention or neurogenic pathologies of the bladder [18].

## 5. Conclusions

Percutaneous stimulation of the tibial nerve to control urinary incontinence has proven to be an effective technique in the different causes that produce urinary incontinence. In addition, it is considered one of the most used methods for overactive bladder syndrome, as well as one of the most promising techniques for the control of the alterations produced in the pelvic floor. A single adverse effect of neuromodulation of the tibial nerve has been observed, being hypoesthesia in one toe for 4 months. Regarding the evaluation of the most appropriate technique for the control of urinary incontinence, both percutaneous tibial nerve stimulation and transcutaneous tibial nerve stimulation have the same efficacy and absence of adverse effects.

## Figures and Tables

**Table 1:** Databases consulted.

Item Criteria	Cochrane	Google Scholar	Medline/Pubmed	Scielo	WOS	Total
Identified	103	132	187	12	77	511
Duplicated	48	83	96	5	31	263
Title	16	19	31	4	14	84
Abstract	12	15	11	3	4	44
Text complete	9	9	7	3	3	32
Valid	8	8	7	2	2	27

## Data Availability

Not applicable.

## Abbreviations

DeCS	Descriptors in Health Sciences
MeSH	Medical Subject Headings
OAB	Overactive bladder syndrome
PTNS	Percutaneous stimulation of the tibial nerve
UI	Urinary incontinence
UUI	Urge urinary incontinence

## Appendix A. Selected Scientific Articles Table

**Authors; Year**	**Type of Study**	**Patients**	**Conclusions**
Marchal C, et al. (2011).	Cut study.	53 patients with posterior tibial nerve stimulation.	It should be noted that posterior tibial nerve stimulation is an appropriate option for the treatment of OAB. In addition, the appropriate point to begin retreatment would be 24 months after the end of therapy.
Burton C, et al. (2012).	Systematic review and meta-analysis.	6 randomized trials and 10 non-randomized prospective studies.	There is a significant improvement in OAB patients receiving treatment with PTNS. This technique is comparable with the effects produced by antimuscarinics, but with better safety regarding side effects. To be able to recommend PTNS as a treatment, it is necessary to obtain long-term data and perform an economic analysis on health, since in the review the included studies only estimated the short-term results after the initial treatment.
Peters KM, et al. (2013)	Prospective study.	35 users.	Performing an average of 1.3 sessions per month, PTNS is a safe option for the treatment of OAB, as well as being durable and effective in the long term for the control of OAB symptoms.
Yoong W, et al. (2013).	Prospective study.	30 women.	Women with PTNS treatment for OAB achieve significant relief of symptoms at 2 years. Therefore, it is considered a second-line treatment, safe and with excellent durability.
Gaziev G, et al. (2013)	Bibliographic review.	32 studies.	The PTNS is a safe and effective technique for patients with OAB; more studies are necessary to be able to evaluate the role of the PTNS in the remaining situations and to be able to evaluate the durability of the long-term treatment.
Moosdorff-Steinhauser HF, et al. (2013)	Systematic Review.	4 RCTs and 6 prospective observational cohort studies.	PTNS is effective for UUI and frequency. However, a higher number of studies with higher quality are needed to improve the evidence on PTNS in nocturia and emergencies, to detail the criteria when selecting patients and the optimal treatment modalities and the effects that may arise long term, in addition to effectiveness in other more pragmatic trials.
Sucar S, et al. (2014)	Bibliographic review.	6 RCT.	Stimulation of the posterior tibial nerve is currently one of the most promising therapeutic techniques for pelvic floor disorders.
Santos E, et al. (2014)	Randomized controlled trial.	24 patients.	PTNS is one of the safe and effective treatment options to treat post-stroke neurogenic OAB.
Priyanka G, et al. (2015)	Narrative review.	-	PTNS and SNM are the most widely used treatments for pelvic floor disorders. PTNS is a minimally invasive process, highly effective in the treatment of IF, OAB and pelvic pain. In addition, it has few side effects, although it is limited, since patients need to visit the office on a weekly basis to receive the sessions. Regarding sacral neuromodulation, it is carried out using an implanted device capable of stimulating the S3 nerve root and, thus, improving pelvic pain, non-obstructive urinary retention (NOUR) and OAB symptoms.
Tubaro A, et al. (2015)	Systematic Review.	-	Botulinum toxin A is recommended as third-line treatment in OAB (USA) and for UUI (USA and Europe) in patients who do not respond positively to drug treatment. Before opting for surgery, it is necessary to assess all the treatment alternatives available for OAB and UUI.
Schneider M, et al. (2015)	Systematic review.	16 studies (4 randomized controlled trials [RCTs], 9 prospective cohort studies, 2 retrospective case series, and 1 case report).	Although the data obtained from RCTs and non-RCTs indicate that TN stimulation may be safe and effective as a treatment for NLUTD, the evidence is insufficient, since the studies are small and non-comparative, with a high risk of confusion and bias.
Zecca C, et al. (2016)	Literature review	7 open prospective studies and 313 patients with multiple sclerosis.	Despite the limitation of the data, PTNS is considered an effective and safe option as a treatment in the management of LUTS for patients with multiple sclerosis.
Manríquez V, et al. (2016)	Randomized controlled trial.	70 patients.	PTNS and ROS show similar benefits in OAB patients after 12 weeks of treatment.
de Wall L, et al. (2017)	Literature review.	-	OAB generally affects adults and children all over the world, which has a great economic and psychological impact. PTNS is a neuromodulation technique used to facilitate storage of the bladder and modulate its function. It is a time-consuming, easy-to-apply and minimally invasive treatment, so implantable devices can be a great solution to solve the long-term financial burden associated with PTNS.
Valles C, et al. (2017)	Retrospective study.	65 patients with urge urinary incontinence refractory to medical treatment.	TPTNS is a well-tolerated and effective treatment for UUIs that do not respond adequately to first-line therapies, and therefore, should be offered earlier in the treatment strategy. It is necessary to carry out new studies to be able to identify the ideal stimulation parameters, the most effective protocols, its long-term efficacy, as well as the correct way to carry it out in patients with a neurogenic basis.
Vecchioli C, et al. (2017)	Randomized controlled trial.	60 women with OAB.	The study shows that PTNS and ES + PFMT are effective in women with OAB, but results are better with PTNS.
Del Río S, et al. (2017)	Prospective study.	200 women diagnosed with OAB.	PTNS is capable of improving OAB symptoms for up to 24 months after completion of treatment. In addition, the first sensation of fullness of the bladder and the diurnal urinary frequency are independent predictors and are of great importance for the success of the PTNS.
Canbaz S, et al. (2017)	Prospective study.	34 patients.	With the results obtained, it has been shown that patients with multiple sclerosis and treated with PTNS can achieve significant durability for 12 months.
Vecchioli C, et al. (2018)	Randomized controlled trial.	105 women with OAB.	All treatments became effective; however, PTNS demonstrated greater effectiveness compared to SS. On the other hand, the combination of both techniques was more effective and with greater durability than the PTNS and SS separately.
Ramírez I, et al. (2019)	Randomized controlled trial.	68 patients (67.6% women), mean age 59.6 years.	This is the first RCT to evaluate the efficacy of TTNS compared to PTNS and shows that there is no inferiority between the two techniques in reducing daytime frequency. In addition, it manages to reduce UUI episodes and improve quality of life, so with the results obtained and its easy application, the prescription of this technique can be increased.
Iyer S, et al. (2019)	Retrospective cohort study.	213 patients.	PTNS is a therapy that can provide both subjective and objective improvements for patients who do not respond to treatment with OAB medication.
Rostaminia G, et al. (2019)	Retrospective Review.	162 women with a mean age of 72.7 ± 11.3 years.	Among women treated for OAB with PTNS, history of anxiety/depression and severe UUI were positive predictors for successful PTNS outcome.
Pincus J, et al. (2019)	Retrospective review.	66 patients.	OAB symptoms remain stable after treatment for 12 sessions per year. However, a history of urogynecological surgery is a negative factor regarding the improvement of patients with OAB symptoms who have received monthly PTNS treatment for 12 months.
Sirls LT, et al. (2019)	Prospective study.	9 women.	CTNS is effective and safe in the treatment of UI and OAB. However, the data are limited.
Alka B, et al. (2020)	Narrative Review.	-	In cases where medication fails to improve symptoms, more invasive treatment options are chosen, such as stimulation of the posterior NT or the sacral nerve or treatments with botulinum toxin A. Scientific professionals recommend the use of percutaneous posterior NT stimulation as third-line treatment, once it has been reviewed by a multidisciplinary team and pharmacological treatment has previously failed.
Fuentes I, et al. (2020)	Prospective observational longitudinal design.	32 women with 58.69 ± 8.96 years.	PTNS using electroacupuncture is an effective treatment for UUI, but its effect decreases over time, even so, the improvement compared to baseline is maintained throughout the follow-up period.
Lashin AM, et al. (2020)	Randomized controlled trial.	57 patients with OAB.	A treatment with PTNS for 6 weeks is more effective and successful than performing sham treatment in patients with OAB. Thus, treatment with PTNS for 6 weeks makes it more cost-effective and more accessible for patients.
